# Acute brain injuries trigger microglia as an additional source of the proteoglycan NG2

**DOI:** 10.1186/s40478-020-01016-2

**Published:** 2020-08-26

**Authors:** Wenhui Huang, Xianshu Bai, Erika Meyer, Anja Scheller

**Affiliations:** 1 Molecular Physiology, Center for Integrative Physiology and Molecular Medicine, University of Saarland, Building 48, 66421 Homburg, Germany; 2 Laboratory of Brain Ischemia and Neuroprotection, Department of Pharmacology and Therapeutics, State University of Maringá, Maringá, CEP-87020900 Brazil

**Keywords:** Microglia, Macrophage, OPCs, Pericytes, NG2, Gliosis, Transgenes, Acute brain injury, MCAO, SWI

## Abstract

NG2 is a type I transmembrane glycoprotein known as chondroitin sulfate proteoglycan 4 (CSPG4). In the healthy central nervous system, NG2 is exclusively expressed by oligodendrocyte progenitor cells and by vasculature pericytes. A large body of immunohistochemical studies showed that under pathological conditions such as acute brain injuries and experimental autoimmune encephalomyelitis (EAE), a number of activated microglia were NG2 immuno-positive, suggesting NG2 expression in these cells. Alternative explanations for the microglial NG2 labeling consider the biochemical properties of NG2 or the phagocytic activity of activated microglia. Reportedly, the transmembrane NG2 proteoglycan can be cleaved by a variety of proteases to deposit the NG2 ectodomain into the extracellular matrix. The ectodomain, however, could also stick to the microglial surface. Since microglia are phagocytic cells engulfing debris of dying cells, it is difficult to identify a genuine expression of NG2. Recent studies showing (1) pericytes giving rise to microglial after stroke, and (2) immune cells of NG2-EYFP knock-in mice lacking NG2 expression in an EAE model generated doubts for the de novo expression of NG2 in microglia after acute brain injuries. In the current study, we took advantage of three knock-in mouse lines (NG2-CreERT2, CX_3_CR_1_-EGFP and NG2-EYFP) to study NG2 expression indicated by transgenic fluorescent proteins in microglia after tMCAO (transient middle cerebral artery occlusion) or cortical stab wound injury (SWI). We provide strong evidence that NG2-expressing cells, including OPCs and pericytes, did not differentiate into microglia after acute brain injuries, whereas activated microglia did express NG2 in a disease-dependent manner. A subset of microglia continuously activated the NG2 gene at least within the first week after tMCAO, whereas within 3 days after SWI a limited number of microglia at the lesion site transiently expressed NG2. Immunohistochemical studies demonstrated that these microglia with NG2 gene activity also synthesized the NG2 protein, suggesting activated microglia as an additional source of the NG2 proteoglycan after acute brain injuries.

**Electronic supplementary material:**

The online version of this article (10.1186/s40478-020-01016-2) contains supplementary material, which is available to authorized users.

## Introduction

Nerve/glia antigen 2 (NG2), encoded by the c*spg4* gene (for simplicity we will use NG2 gene instead of cspg4 gene), is a single membrane-spanning proteoglycan with a large extracellular domain, and a short cytoplasmic tail [43, 57]. In the central nervous system (CNS) of rodents, the earliest NG2 expression was detected in vascular pericytes after embryonic day 10.5 (E10.5) [26, 47]. At E12.5, the NG2 gene starts to get activated in a small population of oligodendrocyte precursor cells (OPCs) in the ventral brain, and subsequently the NG2 protein becomes immuno-detectable in OPCs with increasing proportion from E13.5 to birth [11, 44]. In the postnatal CNS, all OPCs express NG2, therefore they are also termed NG2 glia [23, 42, 49]. Numerous studies using antibody immunostaining and transgenic mouse models revealed that in the healthy CNS, the expression of NG2 is restricted to OPCs and pericytes but cannot be detected in microglia or astrocytes [10, 11, 22–24, 28, 44].

The large extracellular domain of NG2 contains proteolytic sites near the transmembrane domain, allowing the shedding of the NG2 ectodomain by various proteases such as MMP9, MMP13, MMP14, and the α-secretase ADAM10 [51, 57]. Under pathological conditions, glial cells are activated and NG2 expression is greatly enhanced in the injured area, contributing to the formation of the glial scar. Meanwhile, protease level in lesions also increases which could lead to significant deposition of the NG2 ectodomain in the extracellular matrix, therefore blurring the identification of NG2-expressing cells by immunohistochemistry [1, 51, 57].

Previous studies observed NG2 immunoreactivity in a subset of activated Iba1^+^ microglia and/or macrophages (below referred to as microglia for simplicity) in a variety of disease models such as demyelination models induced by lysolecithin or experimental autoimmune encephalomyelitis (EAE) [3, 33, 40, 45], epilepsy model by kainate injection [6], neuroinflammation model by LPS injection [59, 62], or the acute injury models transient middle cerebral artery occlusion (tMCAO) and stab wound injury (SWI) [18, 38, 52, 60]. However, whether microglia could express NG2 upon injury is still under debate, because previous studies were solely based on NG2 immunoreactivity possibly visualizing the shed ectodomain of NG2 after phagocytosis in the inside or after unspecific deposition on the surface of activated microglia [45, 51, 57]. In addition, although NG2 expression largely increased in the acutely injured area, the reported numbers of NG2 immuno-positive microglia varied from study to study. For example, Tanaka’s group showed that in rat brain lesions ~ 65% and ~ 80% of activated microglia displayed NG2 immunoreactivity to a commercial antibody at 7 days post tMCAO and SWI [38, 60], respectively. However, with a house-made antibody Hampton et al., detected only ~ 20% of microglia with NG2 immunoreactivity along the lesion site at 2 days post SWI, and no NG2 immuno-positive microglia at 7 days post injury [18]. These seemingly contradictory results raise doubts that NG2 immuno-positive microglia are NG2-expressing cells in acute brain injury. The situation was further complicated by recent studies suggesting that pericytes (with bona fide expression of NG2) could generate microglia after stroke [46]. In this case, NG2 could be detected as a remnant of a previous cell differentiation event.

On the other hand, the functions of NG2 in diseases were also investigated, though contradictory results appeared among different models. For example, Moransard et al., showed that NG2 detected in macrophages and OPCs was dispensable in an EAE model by using conventional NG2 knockout mice [40]. However, a recent study using NG2-EYFP knock-in (KI) mice in which the expression of EYFP was directly controlled by the endogenous NG2 gene activity demonstrated that NG2 regulated neuroinflammation but was not expressed by immune cells in EAE, suggesting that the contribution of NG2 was derived mainly from OPCs and pericytes [28, 30]. Therefore, it is important to examine whether microglia are activated as sources and/or targets of NG2 in lesions.

In the current study, we used knock-in transgenic mice to investigate the controversial observations in tMCAO and SWI models. We crossbred the myeloid cell-specific GFP-expressing mice (CX_3_CR_1_-EGFP KI) to NG2-CreERT2 KI mice combined with a Rosa26-tdTomato (Ai14) reporter mouse line, thereby the activation of the NG2 gene in EGFP^+^ cells would be traced in terms of tdTomato (tdT) expression upon tamoxifen induced Cre activity [24, 27, 36]. We also used NG2-EYFP KI mice to further confirm the direct gene activity of NG2 after injury [28]. By combining immunohistochemical analysis and transgenic mouse techniques we were able to show that shortly after acute brain injuries a subset of microglia indeed activated the NG2 locus leading to either EYFP expression or tdT recombination. These results suggest that a subset of microglia indeed activates the NG2 gene, leading to an NG2 transcript as well as a successful translation (NG2 protein). However, the time window of NG2 gene activation in microglia was longer lasting after tMCAO (at least the first week after occlusion) than SWI (1–3 days).

## Materials and methods

### Ethics statements

This study was carried out at the University of Saarland in strict accordance with recommendations of European and German guidelines for the welfare of experimental animals. Animal experiments were approved by Saarland state’s “Landesamt für Gesundheit und Verbraucherschutz” in Saarbrücken/Germany (animal license numbers: 71/2010, 65/2013, 36/2016).

### Animals

Mouse breeding and animal experiments were performed in the animal facilities of the University of Saarland. In this study only heterozygous adult mice (2–4 months old) were used to prevent knockin out alleles. Inducible Cre DNA recombinase knock-in mice NG2-CreERT2 were always used in combination with floxed reporter mice (TgH(ROSA26-CAG-fl-stop-sl-tdTomato) = Rosa26-tdT) to show successful recombination (termed NG2-CreERT2xRosa26-tdT mice) [24, 37]. To visualize microglia, the knock-in mouse line CX_3_CR_1_-EGFP was used (TgH(CX_3_CR_1_-EGFP) = CXCR^EGFP^) [27]. NG2-CreERT2xRosa26-tdT mice were crossed to CX_3_CR_1_-EGFP mice to generate triple transgenic mice termed NG2^tdT^xCXCR^EGFP^, simultaneously visualizing NG2-expressing cells and microglia upon tamoxifen administration. For visualization of NG2-expressing cells, the knock-in mouse line NG2-EYFP was analyzed [28].

### Transient middle cerebral artery occlusion

The mice were anesthetized with a mixture of 2% isoflurane and 47.5% O_2_ and 47.5% N_2_O using Harvard Apparatus equipment. Focal cerebral ischemia was induced according to the Koizumi method of middle cerebral artery occlusion (MCAO) [31]. In short, the left common carotid artery (CCA) and the external carotid artery (ECA) were permanently ligated with silk sutures. A silicone-coated filament (Doccol Corp, CA) was introduced through an arteriotomy and advanced into the right internal carotid artery (ICA) until mild resistance was felt, indicating the filament reached the origin of the MCA to occlude the blood flow. After 15 min occlusion the filament was withdrawn and a suture was made around the CCA, to prevent back flow through the arteriotomy.

Mouse body temperature was monitored throughout surgery, occlusion and reperfusion using a rectal thermometer and maintained between 36.5 and 37.5 °C by an adjustable heat plate. After recovery from anesthesia, the mice were kept in their cages with free access to food and water. For three consecutive days the mice received 0.5 mL subcutaneous injection of saline as fluid replacement and intraperitoneal buprenorphine (0.01 µg/30 g body weight, Temgesic, Essex Pharma, Muenchen, Germany) for pain relief. Mice were evaluated for neurological deficits 2 h after the surgery as describe previously [38]. Only mice that developed obvious neurological deficits (e.g. consistently circling to the paretic side or showing reduced resistance to lateral push) were used for the following experiments. In total 27 mice were used for the tMCAO operation, in which five mice died (18.5%) before the end point of the experiment and two mice (7.4%) did not develop obvious neurological deficits.

### Cortical stab wound injury model

Stab wound injury (SWI) was performed in anesthetized mice (ketamine (Ketavet^®^, Pfizer, Germany)/xylazine (Rumpon^®^, Bayer Healthcare, Germany) in 0.9% NaCl (140 mg/10 mg per 1 kg body weight)). The skull was thinned with dental drill laterally 1.5 mm and longitudinally 2 mm from Bregma, followed by a 1 mm deep stab wound made with a scalpel [19].

After SWI the wound was closed and Buprenorphine (0.01 µg/30 g body weight, Temgesic, Essex Pharma, Muenchen, Germany) was injected for anti-pain treatment. In total 17 mice were used for the SWI model and all mice survived after the surgery till the end point of the experiment.

### TTC staining and measurement of the ischemic area

Mice were euthanized and decapitated 24 h after the MCAO operation. Brains were dissected and cut into 2 mm coronal sections. Brain sections were incubated in 2% TTC (2,3,5-triphenyltetrazolium chloride) (Sigma, St. Louis, MO, USA) dissolved in PBS for 10 min at 37 °C. After washing by 1 × PBS, the brain sections were fixed in 4% PFA for 2 h. The images of stained sections were captured under a stereo-microscope (Stemi 2000-C, Zeiss, Göttingen, Germany) equipped with a digital camera (AxioCam ERc 5s, Zeiss, Göttingen, Germany). The ischemic area was determined by measuring the regions that were not stained by TTC using ZEN software (Blue Edition, Zeiss, Oberkochen, Germany).

### Immunohistochemistry

Mouse perfusion, tissue fixation and vibratome slice preparation (40 µm) was performed as described previously [2, 24]. The following primary antibodies were used: polyclonal goat: anti-GFP (1:1000, Rockland, Cat.: 600101215) and anti-Iba1 (1:1000, Abcam, Cat.: ab5076); polyclonal chicken anti-GFP (1:1000, Invitrogen, Cat.: A10262); polyclonal rabbit: anti-NG2 (the extracellular domain) (1:500, Millipore, Cat.: ab5320), anti-Iba1 (1:1000, Wako, Cat.: 01919741), monoclonal rat anti-NG2 (AN2, the extracellular domain) (1:50, gift from Dr. Jacqueline Trotter) [41]. Secondary antibodies from donkey (Alexa488-conjugated anti-chicken, Alexa555/647-conjugated anti-rabbit, Alexa488/647-conjugated anti-goat (all 1:2000)) were from invitrogen, Grand Island NY, USA. Donkey Cy5-conjugated anti-rat IgG (1:500) antibody was purchased from Dianova, Hamburg, Germany. 4′,6‐Diamidino‐2‐phenylindole (DAPI) (0.025 µg/ml final concentration, from AppliChem, Darmstadt, Germany) was added to the secondary antibody dilutions for nuclear staining.

### Tamoxifen-induced gene recombination

Tamoxifen solution was prepared as previously described [23]. To induce reporter expression in NG2^tdT^xCXCR^EGFP^ mice, tamoxifen was intraperitoneally injected (10 µg/ml, 100 µl/10 g body weight) to mice once per day for three consecutive days according to described experiments in each figure.

### Microscopic analysis and quantification

Epifluorescent images were collected by a fully automated slide scanner (AxioScan.Z1, Zeiss, Jena, Germany) equipped with an LED Light Source (Colibri 7, Zeiss, Jena, Germany). The appropriate excitation and emission filters (excitation/emission wavelengths in nm) were set as: 353/465 (DAPI), 488/509 (green), 548/561 (red), and 650/673 (infrared). A Plan-Apochromat 10 ×/0.45 objective for pre-focusing and a Plan-Apochromat 20 ×/0.8 objective for fine focus image acquisition was used. Offline image stitching (8 μm stacks, variance projection) for overviews of brain slices and further analysis was performed using ZEN software (Blue Edition, Zeiss, Oberkochen, Germany).

Confocal images were taken using a laser-scanning microscope (LSM-710, Zeiss) as previously described [23]. Figures presented in this work were modified with Zen 1 software (Black Edition, Zeiss, Oberkochen, Germany).

### Statistical analysis

**Fig. 1:**
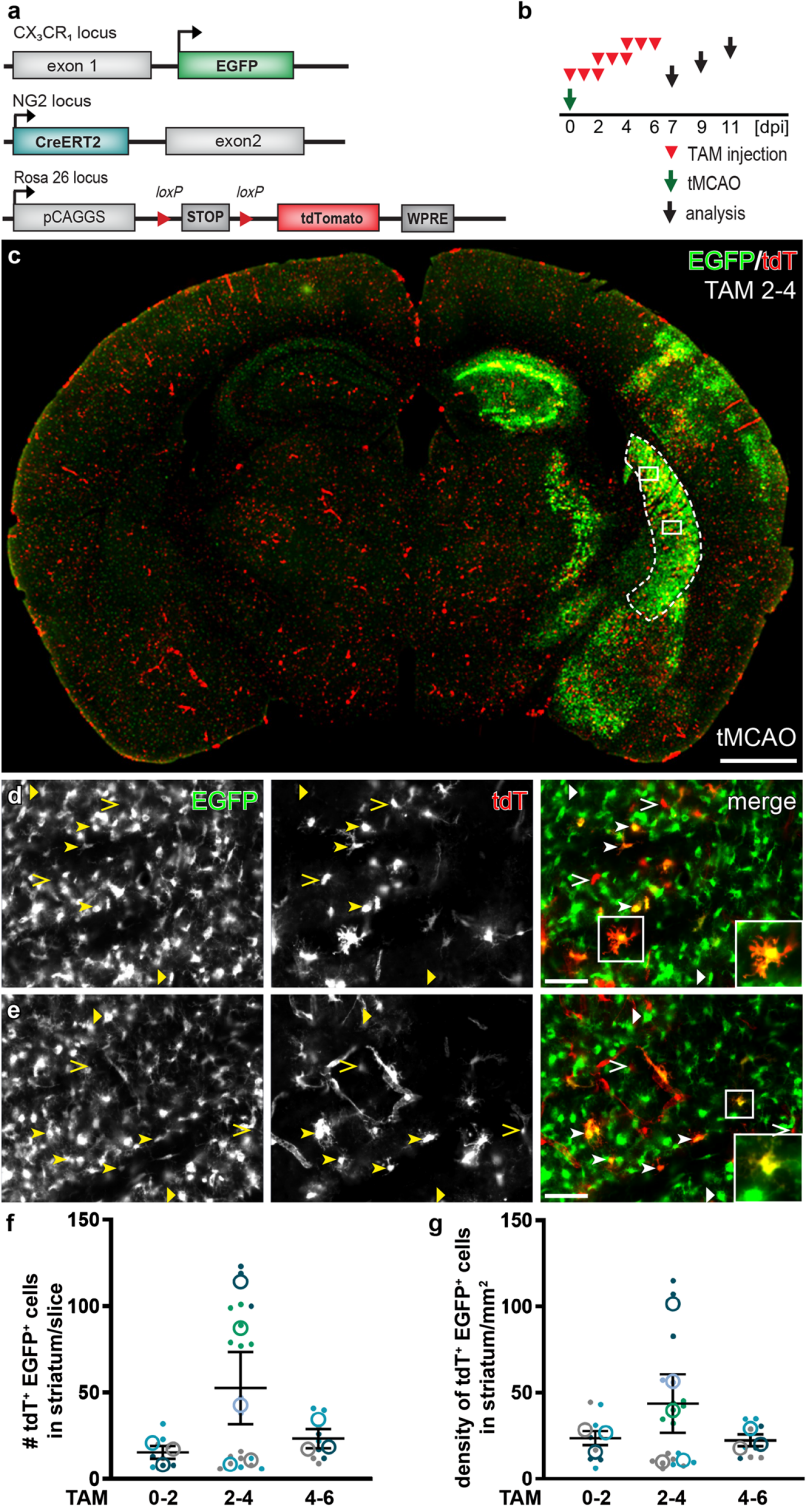
Continuous NG2 gene activation in a subset of microglia after tMCAO. **a** Overview of transgene structures of mouse lines used in tMCAO model. CX_3_CR_1_-EGFP, NG2-CreERT2, and Rosa26-tdTomato mice were crossbred to generate a triple transgenic mouse termed NG2^tdT^xCXCR^EGFP^. **b** NG2^tdT^xCXCR^EGFP^ mice were used for tMCAO. Subsequently injured mice were injected with tamoxifen (TAM) for three consecutive days from 0 to 2 days post injury (0–2 dpi), 2–4 dpi, or 4–6 dpi, and were analyzed at 7, 9, and 11 dpi respectively (7 days after the first day of TAM injection). **c** Overview of a coronal brain section of a NG2^tdT^xCXCR^EGFP^ mouse treated with TAM at 2–4 dpi after tMCAO. EGFP^+^ microglia were drastically activated in the infarct-related area of the brain. **d** and **e** Magnified views from white boxes in **c** showing that in general the expression of EGFP (triangles) and tdT (open arrowheads) were found in distinct cell populations. However, tdT^+^ EGFP^+^ cells (arrowheads) could also be observed. White boxes in **d** and **e** highlighted the morphology of tdT^+^ EGFP^+^ cells representing activated microglia. **f** and **g** Quantification of tdT^+^ EGFP^+^ cells in the infarcted striatum (depicted by the dashed line in **c**) when tamoxifen was injected at different time points, represented as either the numbers of tdT^+^ EGFP^+^ cells per coronal brain slice (**f**) or the density of tdT^+^ EGFP^+^ cells (**g**). The color coding shows the data points per animal (*n* = 3–5 mice indicated as big circles with 2–6 data points (small ones) per mouse). Scale bars = 1000 μm in **c**, and 50 μm in **d** and **e**

For the tMCAO model, 2–6 sections per mouse and three to five mice per group were used. For SWI model, 6–15 sections per mouse and at least five mice per group were analyzed. Data are presented as mean ± SEM of biological replicates (mice). The normal distribution of the data were confirmed by the Shapiro–Wilk test. To compare the number of tdT^+^ EGFP^+^ cells per slice (Figs. 1f, 4f), and the density of tdT^+^ EGFP^+^ cells in Fig. 1g, one-way ANOVA with Tukey’s post hoc multiple comparisons was performed. The levels of significance were set as *P < 0.05; ** < 0.01.

## Results

### NG2 was expressed in a subset of microglia after tMCAO

Previous studies demonstrated the NG2 immunoreactivity in a significant number of microglia within the lesion area after tMCAO. However, those results from immunostaining against NG2 might be attributed to the expression of NG2 in microglia per se, or the shed ectodomain of NG2 after injury. To circumvent the shortcomings of immunohistochemistry for NG2, we took advantages of NG2-CreERT2xRosa26-tdT mice in which NG2-expressing cells could be traced by tdT expression upon tamoxifen administration, and CX_3_CR_1_-EGFP mice in which myeloid cells including microglia and macrophages were visualized by EGFP expression. We crossed NG2-CreERT2xRosa26-tdT mice to CX_3_CR_1_-EGFP mice to generate a new triple transgenic mouse line termed NG2^tdT^xCXCR^EGFP^, allowing visualization of NG2-expressing cells and microglia simultaneously upon induction of Cre activity by tamoxifen (Fig. 1a).

**Fig. 2:**
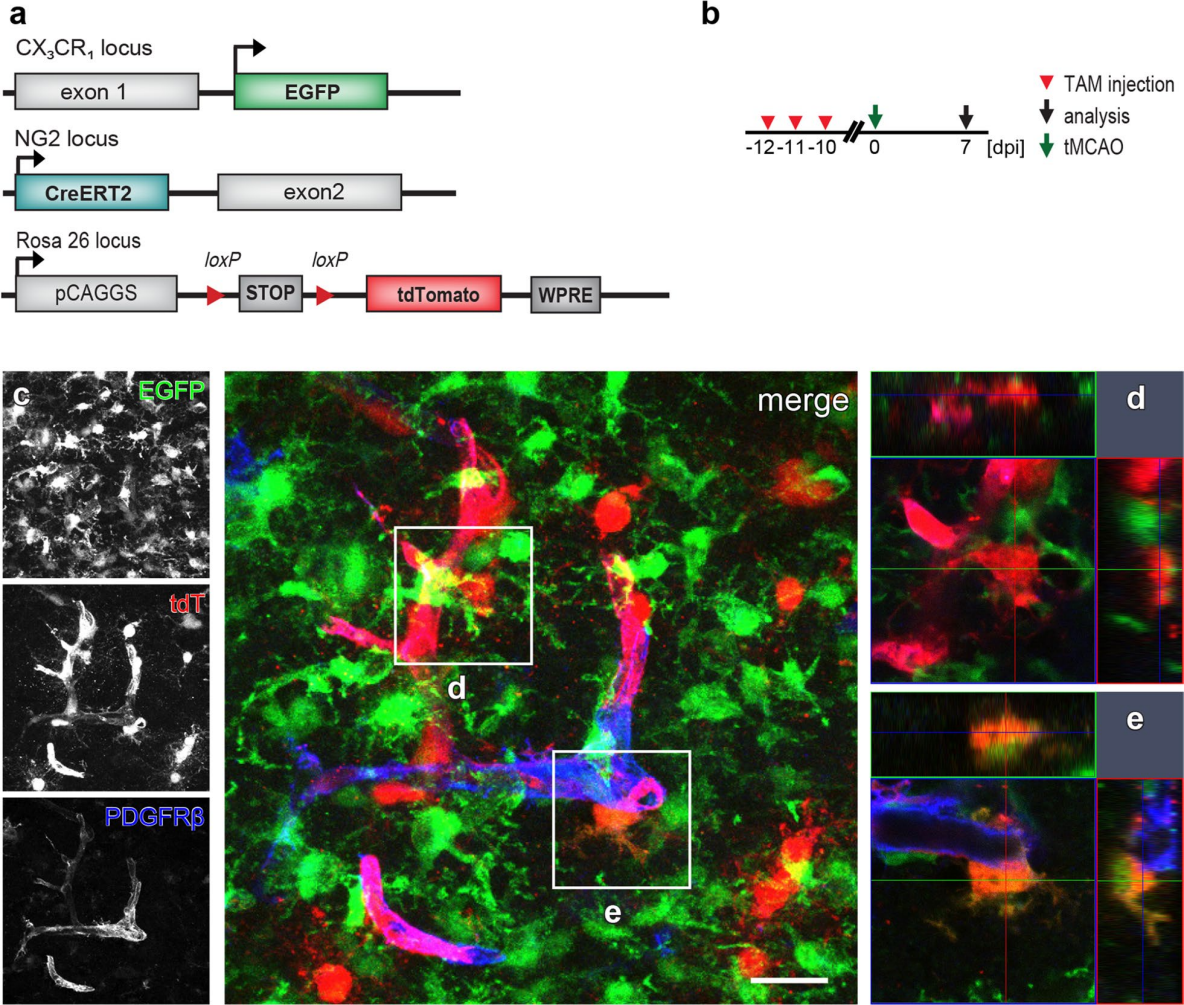
No differentiation of NG2-expressing cells into microglia after tMCAO. **a** Scheme of transgene structures of the triple transgenic mouse line NG2^tdT^xCXCR^EGFP^ used in tMCAO model. **b** Experimental plan: NG2^tdT^xCXCR^EGFP^ mice were occluded (tMCAO) 10 days after the 3-day injection of tamoxifen (TAM) and were analyzed at 7 days post injury (dpi). **c**- **d** Representative confocal images from injured striatum showed that virtually all tdT^+^ cells (including cells in oligodendrocyte lineage and pericytes labeled by PDGFRβ) were not co-expressing EGFP, and intermingled cells could be distinguished by the Z-stack images (**d**, orthogonal view of the corresponding white box in **c**). Very rarely tdT^+^ EGFP^+^ cells (2 cells found in 18 brain slices from three mice, 6 slices per mouse) were detected as indicated in **e** (orthogonal view of the corresponding white box in **c**). Scale bar = 20 μm

**Fig. 3:**
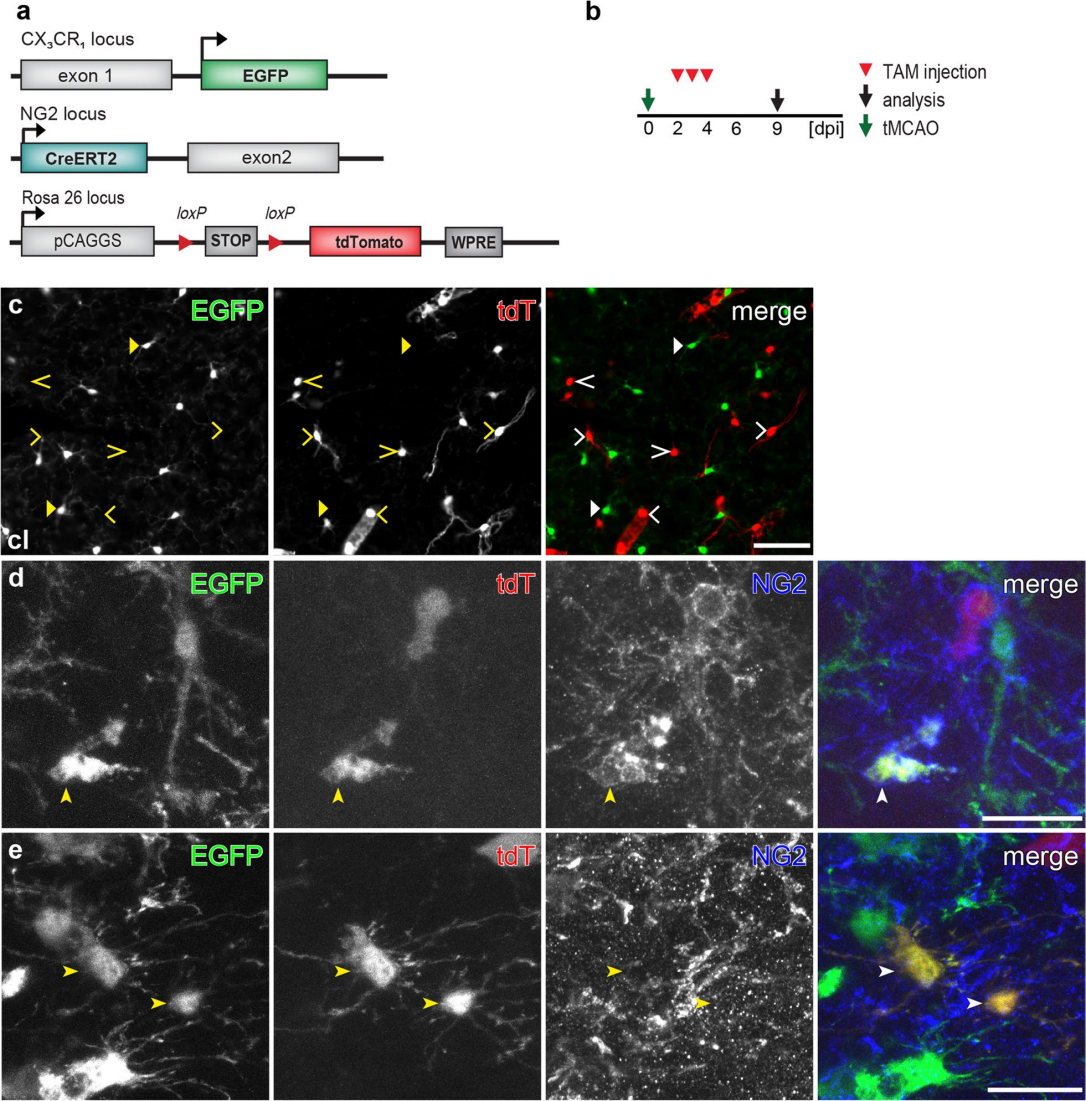
NG2 protein expression in some microglia after tMCAO. **a** Overview of transgene structures of the triple transgenic mouse line NG2^tdT^xCXCR^EGFP^ used in tMCAO model. **b** Experimental plan: NG2^tdT^xCXCR^EGFP^ mice were injected with tamoxifen (TAM) for three consecutive days from 2 to 4 days post injury (dpi) and were analyzed at 9 dpi in the tMCAO model. **c** Micrographs showing EGFP^+^ cells and tdT^+^ cells never overlapped in the contralateral hemisphere. TdT^+^ cells displayed either morphology of oligodendrocyte lineage cells (open arrowheads) or vascular pericytes (open triangle). **d** and **e** Confocal images in the infarct region with tdT^+ ^EGFP^+^ cells immuno-positive (**d**) or immuno-negative (**e**) for NG2. Scale bars = 50 μm in **c**, 20 μm in **d** and **e**

To determine when, if any, EGFP^+^ cells could turn on NG2 gene after tMCAO, we injected adult NG2^tdT^xCXCR^EGFP^ mice with tamoxifen at 0–2, 2–4, or 4–6 days post injury (dpi) for three consecutive days and analyzed at 7 days after the first tamoxifen injection (7, 9, and 11 dpi, respectively) (Fig. 1b). To increase the viability, animals got 15 min tMCAO before reperfusion to induce detectable infarction as previously described [48]. Determined by TTC staining, 25% of the area of coronal brain sections was affected by ischemia 24 h after the operation (Additional file 1: Fig. S1a–c). After tMCAO, we observed highly activated EGFP^+^ cells in terms of increased fluorescent protein expression and cell density in many regions of the infarcted hemisphere of the brain, such as striatum, thalamus, hippocampus and cortex (Fig. 1c, bright green area, Additional file 1: Fig. S1d, e). In parallel, Cre activity induction generated tdT-expressing cells all over the brain, including those infarct-affected regions (Fig. 1c). Although in the gliosis area the majority of tdT^+^ and EGFP^+^ cells still did not overlap, we did detect a small number of EGFP^+^ cells co-expressing tdT whenever Cre activity was induced after tMCAO. Microglia are known to display numerous morphological changes upon stimulation [29]. We noticed that those tdT^+^ EGFP^+^ cells also possessed diverse morphologies such as ramified, amoeboid, or round shapes, representing activated microglia and/or macrophages (Figs. 1d, e, 3d, e) [14, 34]. In contrast, virtually no tdT^+^ EGFP^+^ cells were found in the injured regions when tMCAO was performed 10 days after tamoxifen administration (Fig. 2), suggesting tdT^+^ EGFP^+^ cells resulted from acute NG2 gene activity in triggered microglia rather than from the differentiation of recombined NG2-expressing cells. We therefore conclude that a small population of microglia activated the NG2 gene after tMCAO.

To investigate the dynamic activation of the NG2 gene in microglia after tMCAO, we quantified the numbers of tdT^+^ EGFP^+^ cells found in the ipsilateral striatum, the most affected region, from different time points of tamoxifen treatment. When Cre activity was induced at 0–2 dpi, 15 ± 7 tdT^+^ EGFP^+^ cells could be found in the striatum per brain slice. The number of tdT^+^ EGFP^+^ cells increased to 50 ± 21 per slice and then fell back to 23 ± 6 per slice when tamoxifen was injected at 2–4 and 4–6 dpi, respectively (Fig. 1f). The corresponding density of those detected tdT^+^ EGFP^+^ cells also showed a similar trend along the time line of tamoxifen administration (24 ± 4, 43 ± 17, and 22 ± 3 cells/mm^2^ from striatum treated with tamoxifen at 0–2, 2–4 and 4–6 dpi, respectively) (Fig. 1g, Additional file 1: Fig. S1f, g). In the contralateral hemisphere, no tdT^+^ cells with EGFP expression were found, indicating microglial NG2 activity was triggered by the injury (Fig. 3a–c). Therefore, activation of the NG2 gene occurred constantly in a small population of microglia after tMCAO. Furthermore, immunohistochemical experiments with antibodies against the extracellular domain of NG2 demonstrated that many of those tdT^+^ EGFP^+^ cells were immuno-positive for NG2 (Fig. 3d, e), suggesting that those tdT^+^ microglia not only activated NG2 promoter but also synthesized NG2 protein.

Taken together, our current results from NG2^tdT^xCXCR^EGFP^ mice provide strong evidence for an acute NG2 expression in a subset of microglia in the infarct-affected regions, at least within the first week after tMCAO.

### SWI leads to the transient activation of the NG2 gene in a subset of microglia

**Fig. 4:**
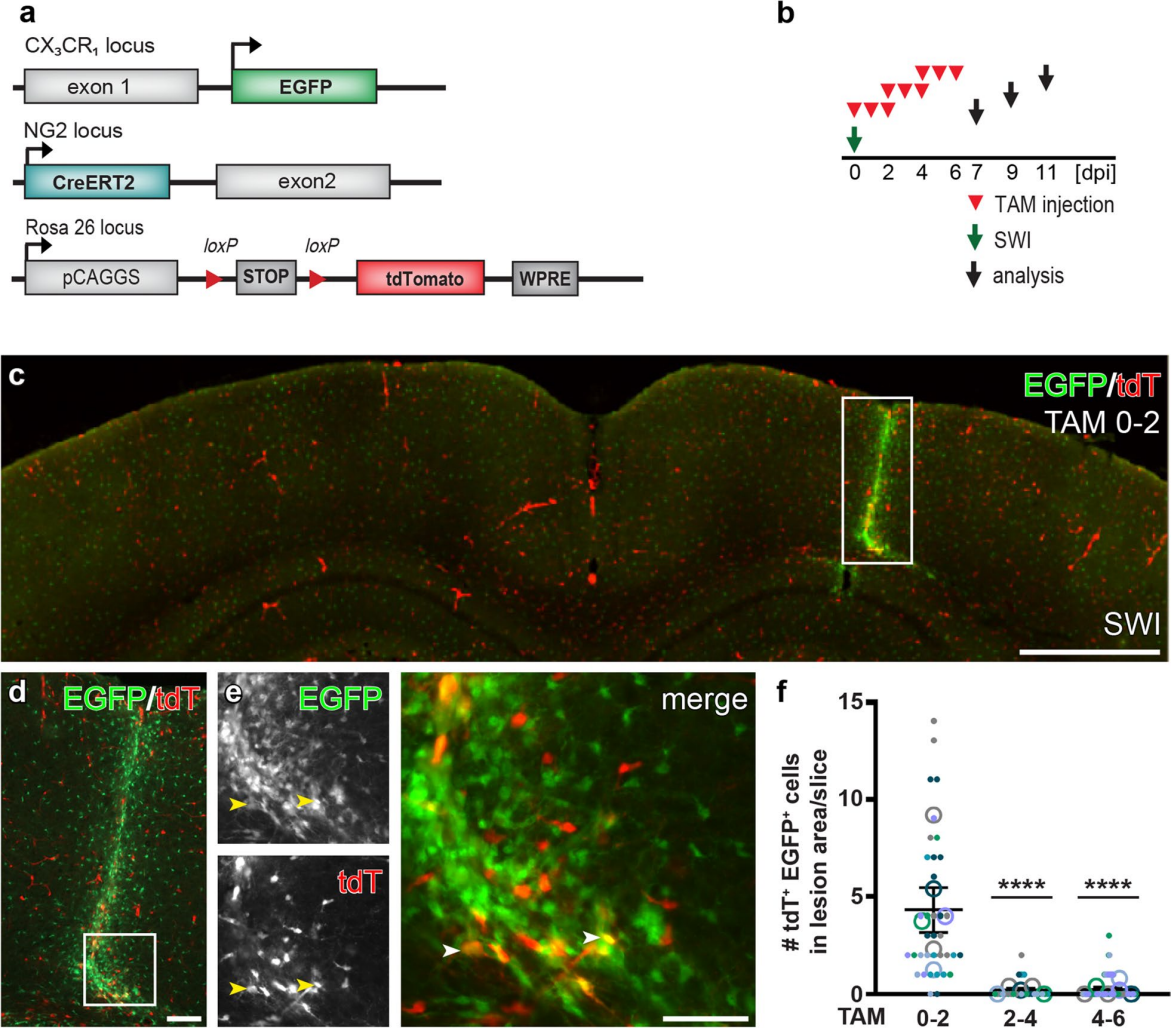
Transient activation of the NG2 gene upon SWI in a subset of microglia. **a** Schematic of transgene structures of NG2^tdT^xCXCR^EGFP^ mice used in SWI model. **b** Experimental plan: Subsequently after SWI, injured NG2^tdT^xCXCR^EGFP^ mice were injected with tamoxifen (TAM) for three consecutive days from 0 to 2 days post injury (0–2 dpi), 2–4 dpi, or 4–6 dpi, and were analyzed at 7, 9, and 11 dpi respectively. **c** Overview of a coronal brain section of a NG2^tdT^xCXCR^EGFP^ mouse at 7 dpi treated with TAM at 0–2 dpi. Along the injury site in the cerebral cortex, activation of EGFP^+^ microglia were detected in terms of increased cell density and EGFP expression. **d** Injured area (white box in **c**) shown in higher magnification. **e** Magnified view from white box in d showing a few tdT^+^ EGFP^+^ cells (arrowheads) at the lesion site. **f** Numbers of tdT^+^ EGFP^+^ cells found per coronal brain slice when tamoxifen was injected at different time points after SWI. The color coding shows the data points per animal (*n* = 3–5 mice indicated as big circles with 5–15 data points (small ones) per mouse). Scale bars = 1000 μm in **c**, 100 μm in **d**, and 50 μm in **e**

**Fig. 5:**
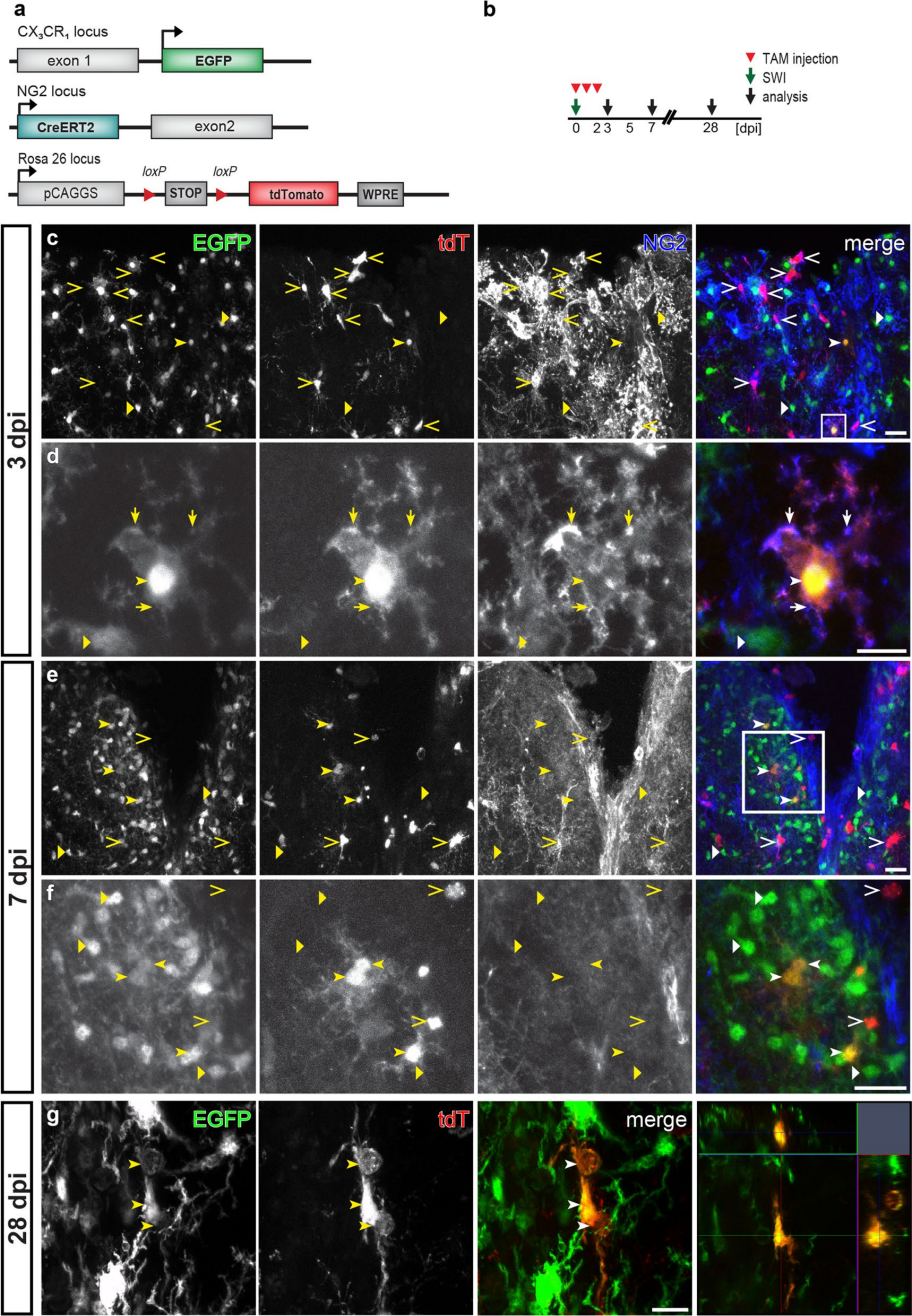
NG2 gene-activated microglia with NG2 protein expression within short time after SWI. **a** Overview of transgene structures of NG2^tdT^xCXCR^EGFP^ mice used in SWI model. **b** Experimental design: After SWI, NG2^tdT^xCXCR^EGFP^ mice were injected with TAM from 0 to 2 dpi, and analyzed at 3, 7, or 28 dpi. **c**–**f** Confocal images showing NG2 immunoreactivity in tdT^+^, EGFP^+^ cells at the lesion site at 3 dpi (**c**, **d**), and 7 dpi (**e**, **f**). EGFP (triangles) and tdT (open arrowheads) were mostly expressed in different cell types, with exceptions of a few tdT^+^ EGFP^+^ cells detected at the lesion site (arrowheads). The immunoreactivity to NG2 was detected in some tdT^+^ EGFP^+^ cells (arrows indicate the NG2 positive processes) at 3 dpi (**d**, magnifying the white box in **c**) but undetectable at 7 dpi (**f**, magnifying the white box in **e**), whereas most cells expressing only tdT (arrowheads) were always NG2 immunopositive with some exceptions indicating their differentiation into mature oligodendrocytes. **g** Confocal images showing a tdT^+^ EGFP^+^ cell found at 28 dpi, with the orthogonal view (right image). Scale bars = 20 μm in **c**, **e**, **f**, and 10 μm in **d**, **g**

Unlike tMCAO, SWI is type of acute brain injury with direct bleeding into the parenchyma. To investigate whether microglia also acquire a similar dynamic activation of the NG2 gene after cortical SWI, we used the NG2^tdT^xCXCR^EGFP^ mice with the same tamoxifen induction protocols as used in the tMCAO model (Fig. 4a, b). SWI triggered a substantial activation of microglia along the lesion site (Fig. 4c, d, bright green area). Tamoxifen treatment could always induce recombination of the tdT reporter over the whole brain including the injured area. Like in the tMCAO model, no tdT expression was detected in EGFP^+^ cells outside the injured area. However, we could always observe a small number of tdT^+^ EGFP^+^ cells (~ 5 cells per brain slice) at the lesion site (within 300 µm of the stab wound) when tamoxifen was administered at 0–2 dpi (Figs. 4e, f, 5). The number of tdT^+^ EGFP^+^ cells quickly decreased to less than ~ 1 cell/per brain slice when tamoxifen was given later at 2–4 or 4–6 dpi (Fig. 4f). In addition, without tamoxifen we only observed very sporadic tdT^+^ cells even at the lesion site that never co-expressed EGFP, indicating tdT^+^ EGFP^+^ cells were not resulted from injury-induced spontaneous recombination (Additional file 1: Fig. S2). When Cre activity was induced 70 days before SWI, we detected a number of tdT^+^ cells without any EGFP expression at the lesion site at 3 or 14 dpi, suggesting that NG2^+^ cells did not differentiate into microglia (Additional file 1: Fig. S2). Notably, whenever tamoxifen was administered, we could detect cell debris of tdT^+^ cells being phagocytosed by EGFP^+^ microglia after SWI (Additional file 1: Fig. S3, tdT^+^ particles wrapped by EGFP^+^ cells), which could be easily distinguished from the EGFP^+^ cells co-expressing tdT by their morphology. Therefore, the present results suggest that a small number of microglia at the lesion site transiently activated their NG2 gene shortly after SWI.

**Fig. 6:**
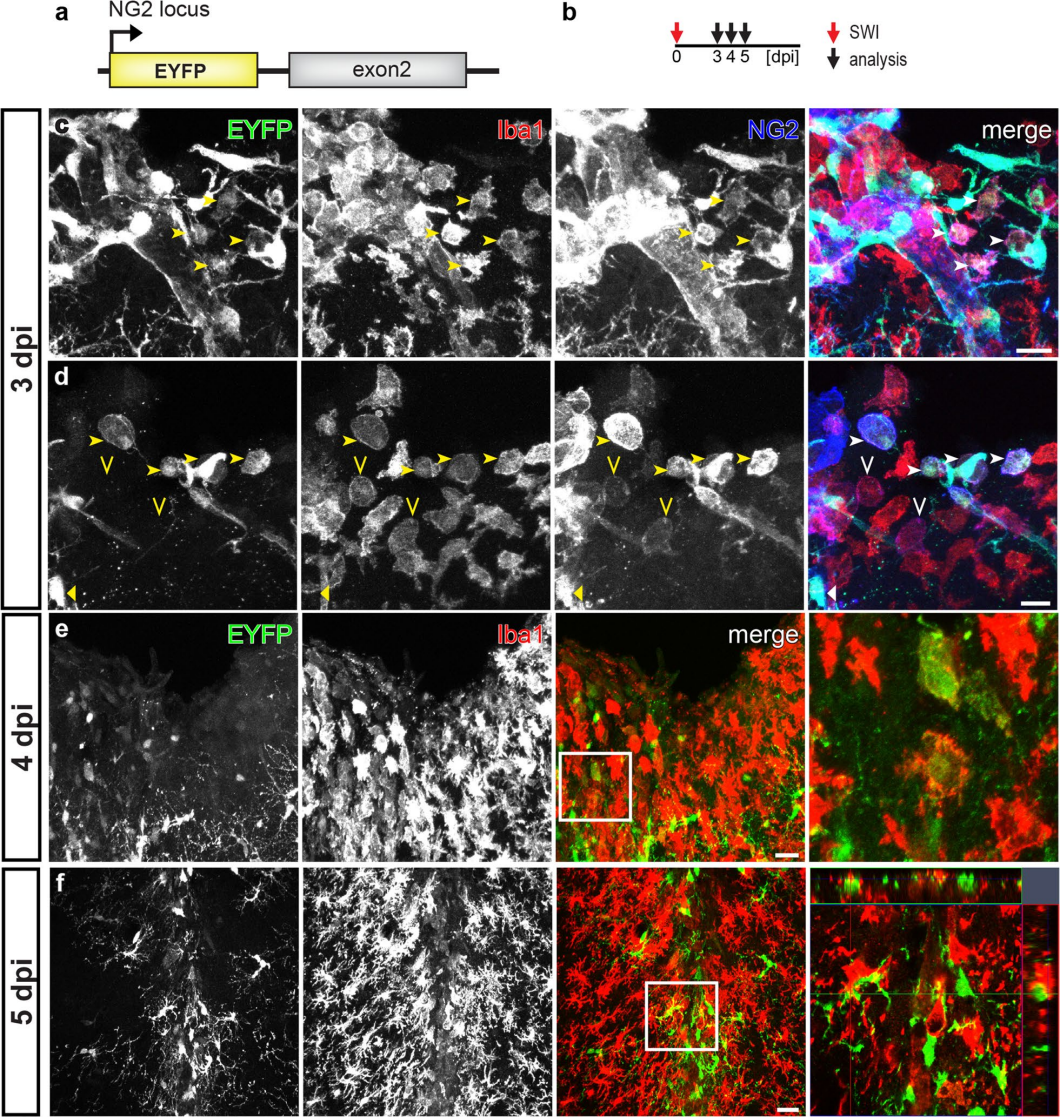
Few microglia in NG2-EYFP mice with NG2 promotor activity after SWI. **a** Schematic of transgene structure of NG2-EYFP mice used in SWI model. **b** Experimental plan: NG2-EYFP mice were analyzed at 3, 4, or 5 dpi (SWI). **c** and **d** Confocal micrographs showing EYFP^+^, Iba1^+^, and NG2^+^ cells at the lesion site at 3 dpi. A few NG2^+^ Iba1^+^ cells could be detected at the lesion site with (arrowheads in **c** and **d**) or without (open arrowheads in **d**) expression of EYFP. **e** and **f** Confocal images showing EYFP and Iba1 immuno-positive cells at the injured area at 4 (**e**) and 5 (**f**) dpi. EYFP^+^ Iba1^+^ cells could be rarely observed at 4 dpi (**e**, white box showing an example magnified in the right image), and were almost undetectable at 5 dpi (**g**, white box was shown as orthogonal view in the right image). Scale bars = 20 μm

To determine whether those NG2 gene-active microglia also produce the NG2 protein after SWI, we induced Cre activity at 0–2 dpi and performed immunohistochemistry for NG2 at 3, 7 or 28 dpi (Fig. 5a, b). In line with the results from the study for dynamic activation of NG2 gene, we only detected NG2 immunoreactivity on tdT^+^ EGFP^+^ cells at 3 dpi, but never at 7 or 28 dpi (Fig. 5c–g). To further confirm the NG2 expression in activated microglia after SWI, we took advantage of NG2-EYFP knock-in mice, which demonstrate endogenous NG2 gene activation as EYFP expression. We performed immunostaining against Iba1 (a marker for microglia) and NG2 at 3, 4, or 5 day after SWI to seek microglia with EYFP and NG2 expression (Fig. 6a, b). At 3 dpi, we detected few NG2^+^ Iba1^+^ cells expressing EYFP at the lesion site (Fig. 6c, d, arrowheads). These EYFP^+^ Iba1^+^ cells showed amoeboid or round shape, representing activated microglia or macrophage-like morphology. In addition, we also observed NG2^+^ Iba1^+^ cells without EYFP expression (Fig. 6d, open arrowheads), possibly due to the degradation of EYFP after the transient activation of the NG2 gene. At 4 dpi, it became rather difficult to find Iba1^+^ cells co-expressing EYFP (Fig. 6e). One day later (5 dpi) no overlay between EYFP-expressing cells and Iba1 immunoreactivity could be observed anymore (Fig. 6f). Regarding the half-life of jelly fish proteins (EGFP: ~ 26 h) [9], current studies of NG2-EYFP mice suggested a time window of 1–3 days for the NG2 promoter activation in microglia after SWI, in line with results from NG2^tdT^xCXCR^EGFP^ mice.

## Discussion

Several previous studies detected NG2 immunoreactivity on microglia or macrophages in a variety of disease models. However, the existence of NG2-expressing microglia was still debated. Main reasons were alternative explanations inherent to the biochemistry of NG2 itself and its cell biology. Both of which are difficult to be addressed by immunohistochemistry only: (1) the shed ectodomain of NG2 and a possible deposition at the lesion site, (2) differentiation of pericytes into microglia with residual NG2 protein, and (3) microglial phagocytotic activity of dying NG2^+^ cells [45, 46, 51, 57]. Indeed, microglia could engulf myelin debris upon injury [5, 13, 50, 58]. Furthermore, a recent study using NG2-EYFP mice found no NG2-expressing immune cells in an EAE model, raising doubts for NG2-expression by microglia in other disease models [30]. In the present study, we took advantage of the direct fluorescent protein expression in the triple transgenic mouse line NG2^tdT^xCXCR^EGFP^ to circumvent the shortcomings of immunohistochemical studies of NG2 expression in microglia, by temporally controlling the induction of Cre DNA recombinase activity.

When Cre activity was induced before tMCAO or SWI, we virtually never detected tdT^+^ EGFP^+^ cells, indicating that NG2-expressing cells, including NG2 glia and pericytes, do not differentiate into microglia. Our results were in line with previous studies demonstrating that NG2 glia/OPCs did not generate microglia, as well as a recent study using Tbx18-CreERT2 mice showing that pericytes did not behave as stem cells to differentiate into other cells types in a variety of disease models including SWI [16, 42]. Notably, some studies suggested that pericytes might give rise to microglia after injury. For example, RGS5 (regulator of G-protein signaling 5) is another marker of pericytes. In a permanent MCAO stroke model using RGS5-GFP mice GFP^+^ cells were immuno-positive for microglial markers [46]. However, microglia were reported to transiently express non-microglial genes upon activation [53], therefore, it was possible that the RGS-5 promoter was somehow induced in microglia after stroke, enabling the GFP expression. On the other hand, we cannot rule out that differences of disease models triggered pericytes to behave differently. Nevertheless, our current results showing NG2-expressing cells did not generate microglia further confirmed that tdT^+^ EGFP^+^ cells being detected when Cre activity was induced after injuries were indeed due to the activation of the NG2 gene in a subset of microglia.

Transient MCAO induced long-lasting brain damage, while after cortical SWI the wound healed quickly with reduced glia activation [12, 20, 35, 39]. With NG2^tdT^xCXCR^EGFP^ mice, we were able to show that the generation and the time frame of appearance of NG2-expressing microglia also differed in those two injury models by inducing Cre activity at different time points. In tMCAO, a small population of EGFP^+^ microglia continuously activated the NG2 gene in terms of tdT expression at least in the first post-lesion week, whereas microglial NG2 gene activity could only be detected at the lesion site shortly after SWI. In addition, we observed more tdT^+^EGFP^+^ cells when NG2^tdT^xCXCR^EGFP^ mice were treated with tamoxifen at 2–4 days post tMCAO, coincident with previous studies demonstrating the MCAO-induced damage developed progressively to reach the peak at 2–3 dpi [20, 35]. Therefore, the present results suggest that microglial NG2 gene activation is dependent on the type and severity of injury.

Our immunohistochemical experiments detected a subset of tdT^+^ EGFP^+^ cells with NG2 immunoreactivity in both injury models, strongly suggesting that those cells also express NG2 protein. After SWI, the detectable NG2 immunoreactivity in microglia in NG2^tdT^xCXCR^EGFP^ mice or in NG2-EYFP mice all showed up within the first 3 days, generally consistent with the NG2 gene activity revealed by reporter gene expression. However, in NG2^tdT^xCXCR^EGFP^ mice we also found some tdT^+^ EGFP^+^ cells without NG2 immunoreactivity in the tMCAO model which triggered continuous activation of NG2 gene as well as at 3 dpi in the SWI model. Because Cre mediated recombination generated permanent tdT expression, those observations of NG2 immuno-negative tdT^+^ EGFP^+^ cells could be interpreted in such way, that the NG2 protein had been already degraded. On the other hand, it has been reported that some cortical neurons in the postnatal brain could acquire slight NG2 gene activity leading to the reporter recombination in NG2-CreERT2xRosa26-tdT mice, but without inducing the expression of NG2 protein [24]. Therefore, we could also conceive that some microglia possessed moderate NG2 gene activity after injury which enabled only the expression of Cre rather than endogenous NG2 protein. Nevertheless, although the dynamic regulation of NG2 protein in microglia remains to be elucidated, our current results provide strong evidence that microglia are one of the sources of NG2 protein after acute brain injuries.

Although we observed significant numbers of tdT^+^ EGFP^+^ cells in our tMCAO model, the number of tdT^+^ EGFP^+^ cells was still lower than numbers of NG2^+^ microglia found in previous studies in rat (~ 60%) [38, 55]. The brain damage increased with the occlusion period in the tMCAO model, which might account for the discrepancy detected in those studies (15 and 90 min in the present and previous studies, respectively) [38, 55]. Of note, in SWI models the number of tdT^+^ EGFP^+^ cells found in the present mouse study was also much lower than the number of microglia with NG2 immunoreactivity found in rat (at least 20%) [17, 60]. Therefore, we cannot rule out that the species difference regarding NG2 gene activity contributed to the discrepancy among those studies as well. The biological significance of the NG2 proteoglycan generated from microglia in our models remains to be determined. Further experiments combining NG2 floxed mice [8] and microglia-Cre expressing mice (such as CX_3_CR_1_-Cre [61]), however, will facilitate the study of functions of the NG2 expressed by microglia.

The NG2 immunoreactivity in microglia is not restricted to tMCAO and SWI, as it was studied by us, it was also reported in other rodent models of CNS trauma such as spinal cord injury (SCI) or LPS-induced inflammation [25, 62]. Moreover, several studies suggest that the NG2 proteoglycan is involved in axon growth and inflammatory response [21, 32, 56], and transplantation of NG2^+^ microglia ameliorated ischemic damage in rat brain [52]. On the other hand, previous immunohistochemical studies in human specimen suggested a similar NG2 expression pattern as in rodents. For example, NG2 could be detected in OPCs and pericytes in the healthy human CNS and was also found upregulated under pathological conditions [4, 54]. Furthermore, NG2 was immuno-detectable in microglia/macrophages in samples from patients who encountered acute CNS injuries such as SCI or stroke, though the functions were not determined [7, 52]. Therefore, combined with other techniques such as genetic cell ablation [15], NG2^tdT^xCXCR^EGFP^ mice would be a valuable tool for the mechanistic study of the generation and function of NG2-expressing microglia, as well as the contribution of NG2 derived from distinct cell populations in different diseases.

## Supplementary information

**Additional file 1: Supplementary Figure S1**. Assessment of infarct-affected area in the brain. **Supplementary Figure S2**. After SWI tdT^+^ EGFP^+^ cells were not derived from spontaneous recombination or pre-existing NG2-expressing cells. **Supplementary Figure S3**. Microglial phagocytosis of dying tdT^+^ cells.

## Abbreviations

CNS: Central nervous system
dpi: Days post injury
EAE: Experimental autoimmune encephalomyelitis
NG2: Nerve/glia antigen-2
OPC: Oligodendrocyte precursor cell
Rosa26-tdT: Rosa26Actin^fl^STOP^fl^tdTomato
SWI: Stab wound injury
tam: Tamoxifen
tdT: tdTomato
tMCAO: Transient middle cerebral artery occlusion
