# Supplementary material for: Acute brain injuries trigger microglia as an additional source of the proteoglycan NG2
Source: Acta Neuropathol Commun. 2020 Aug 26;8:146. doi: 10.1186/s40478-020-01016-2 (PMC7449013; doi:10.1186/s40478-020-01016-2)
Supplement: Supplementary file 1 — Additional file 1: Supplementary Figure S1. Assessment of infarct-affected area in the brain. Supplementary Figure S2. After SWI tdT+ EGFP+ cells were not derived from spontaneous recombination or pre-existing NG2-expressing cells. Supplementary Figure S3. Microglial phagocytosis of dying tdT+ cells. [file 40478_2020_1016_MOESM1_ESM.pdf]

Supplementary Figures

Acute brain injuries trigger microglia as an additional source of the proteoglycan NG2

Wenhui Huang[1]*, Xianshu Bai[1], Erika Meyer[1, 2] and Anja Scheller[1]*

1. Molecular Physiology, Center for Integrative Physiology and Molecular Medicine, University of Saarland, D-66421 Homburg, Germany
2. Laboratory of Brain Ischemia and Neuroprotection, Department of Pharmacology and Therapeutics, State University of Maringá, CEP-87020900, Maringá, Brazil

(*) To whom correspondence should be addressed.

Wenhui Huang:

Molecular Physiology, Center for Integrative Physiology and Molecular Medicine, Building 48, University of Saarland, 66421 Homburg, Germany

Email: wenhui.huang@uks.eu
Phone: +49 6841 1616 446

Anja Scheller:

Molecular Physiology, Center for Integrative Physiology and Molecular Medicine, Building 48, University of Saarland, 66421 Homburg, Germany

Email: anja.scheller@uks.eu
Phone: +49 6841 1616 460
+49 6841 1616 443

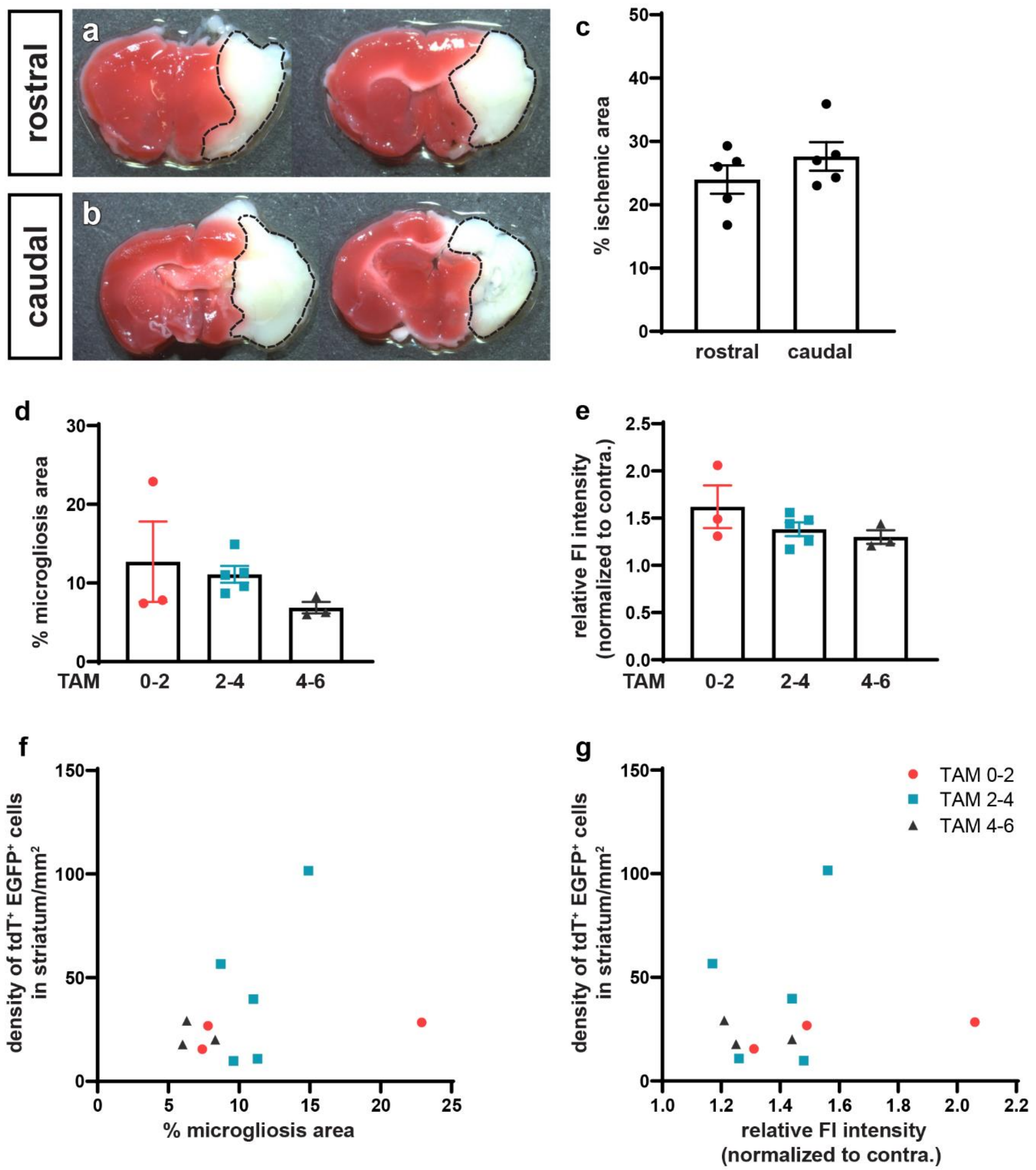

Supplementary Figure S1. Assessment of infarct-affected area in the brain.

a and b Images showing the TTC staining of coronal brain sections at rostral (**a**) and caudal (**b**) level 24 h after tMCAO. **c** Proportion of ischemic areas (as indicated in **a** and **b** by dashed line, n = 5 mice). **d and e** Bar graphs quantify the percentage of microgliosis (as area) indicated by enhanced EGFP expression (**d**), and the relative fluorescent intensity (FI) of the ipsilateral brain normalized to the contralateral part of coronal sections (**e**) from mice in Figure 1f and 1g. **f and g** Data points of the density of tdT^+ $EGFP^+$ cells in striatum shown in Figure 1g were plotted with the corresponding data points in **d** and **e**. No obvious correlation between the density of tdT^+ $EGFP^+$ cells and the extent of microgliosis from each experimental time points could be detected.

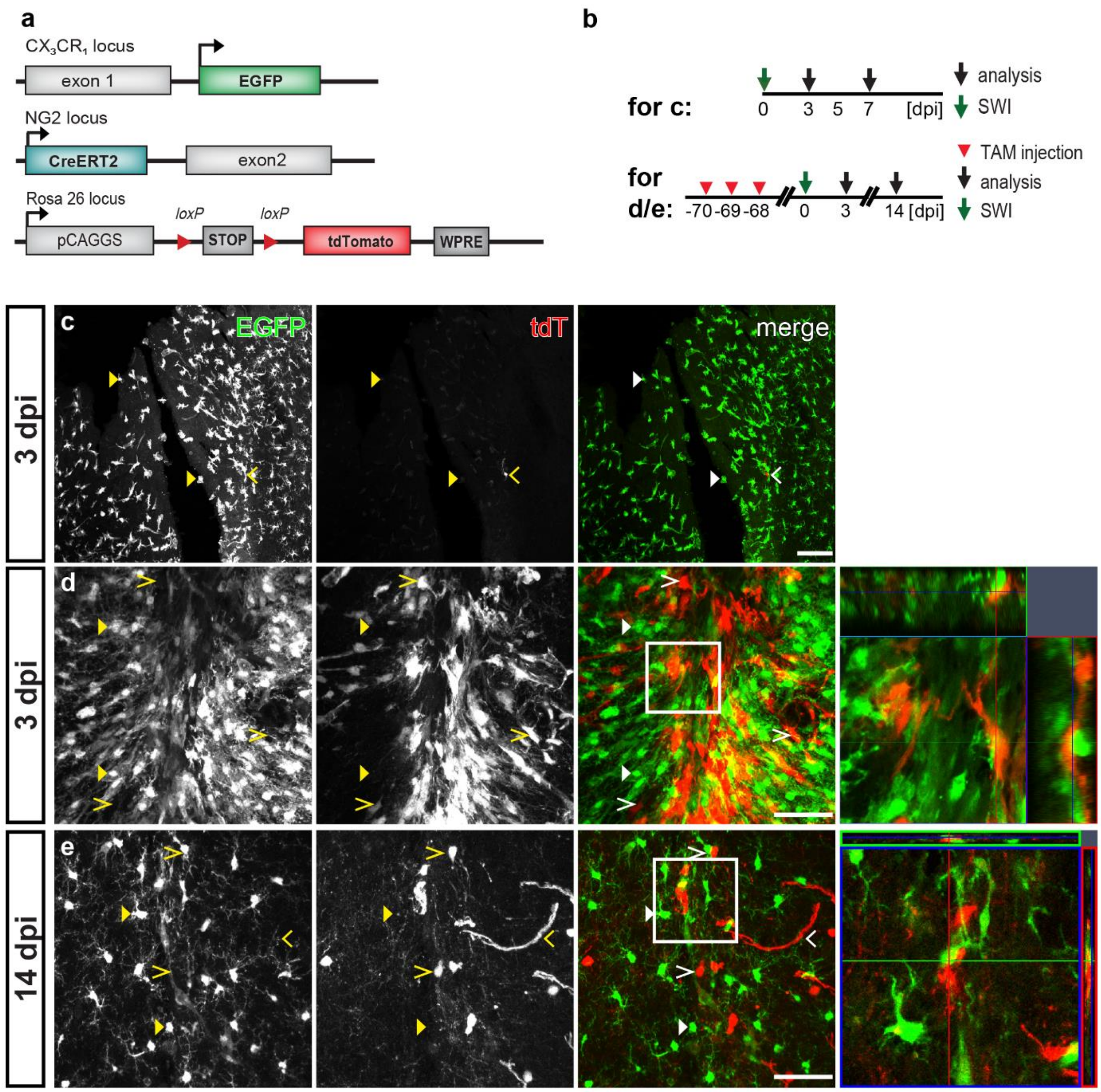

Supplementary Figure S2. After SWI tdT$^+$ EGFP$^+$ cells were not derived from spontaneous recombination or pre-existing NG2-expressing cells.

a Overview of transgene structures of NG2^{tdT}xCXCR^{EGFP} mice used in SWI model. **b** NG2^{tdT}xCXCR^{EGFP} mice were used for SWI and analyzed at 3 or 7 dpi, without tamoxifen treatment (for **c**). In addition, NG2^{tdT}xCXCR^{EGFP} mice were also injected with tamoxifen 70 days prior to SWI and analyzed at 3 or 14 dpi (for **d**/**e**). **c** Micrographs showing only sporadic tdT expression at the lesion site and never in EGFP$^+$ cells without tamoxifen induced recombination. **d and e** Confocal images showing EGFP$^+$ (triangles), tdT$^+$ glial cells (open arrowheads), and tdT$^+$ pericytes (open triangles) at the lesion sites at 3 (**d**) or 14 (**e**) dpi. EGFP and tdT signals were never co-localized as shown by orthogonal views of the white boxes, indicating that tdT$^+$ cells did not differentiate into EGFP$^+$ cells. Scale bars = 20 μm.

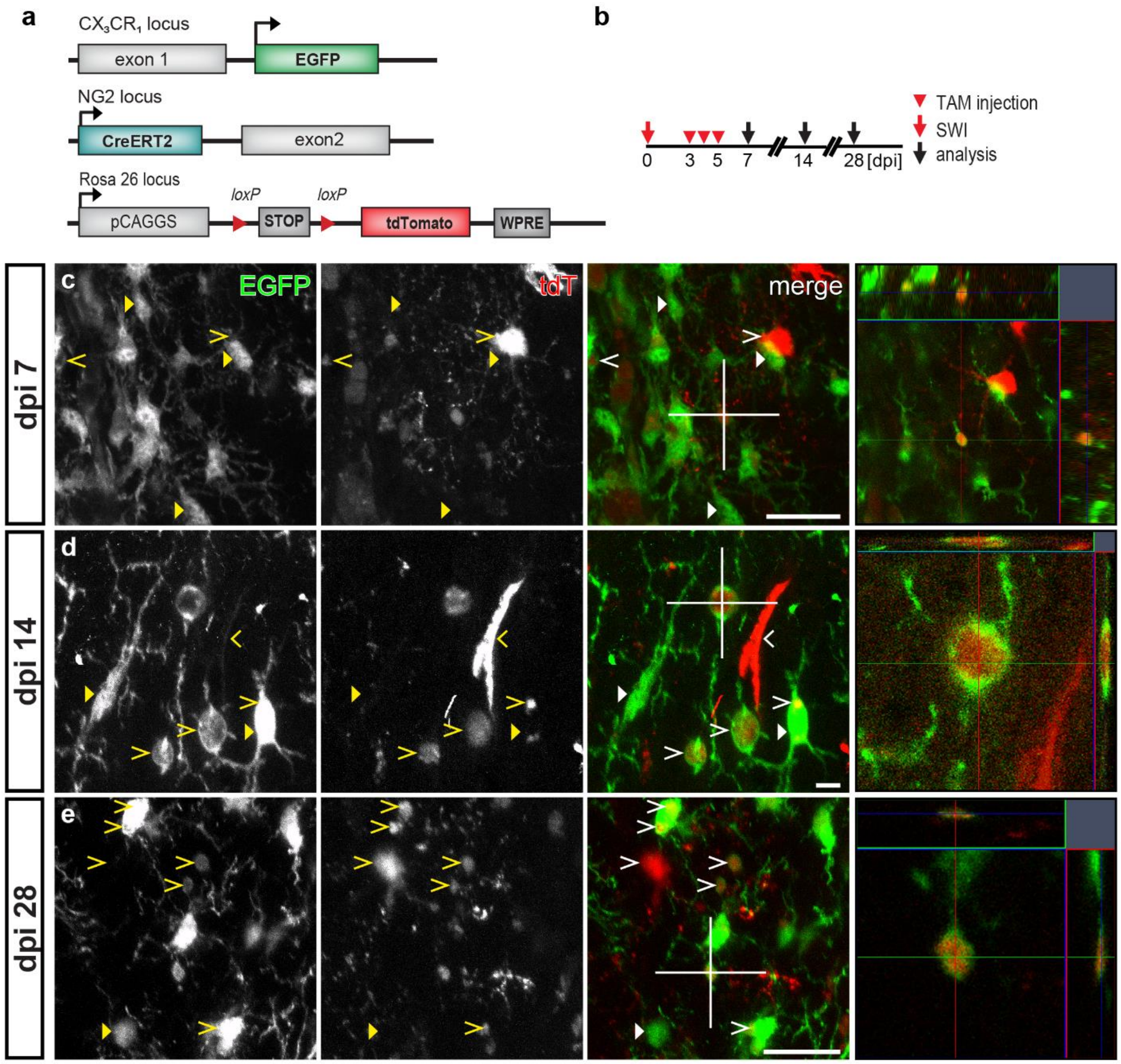

Supplementary Figure S3. Microglial phagocytosis of dying tdT$^+$ cells.

a Overview of transgene structures of NG2^{tdT}xCXCR^{EGFP} mice used in SWI model. **b** After SWI, NG2^{tdT}xCXCR^{EGFP} mice were injected with TAM from 3-5 dpi to induce Cre activity, and subsequently analyzed at 7, 14, or 28 dpi. **c-e** Confocal images showing EGFP$^+$ (triangles) and tdT$^+$ cells (open triangles) along the lesion site of NG2^{tdT}xCXCR^{EGFP} mice at 7 (**c**), 14 (**d**), and 28 (**e**) dpi. Notably, some condensed tdT$^+$ particles trapped in EGFP$^+$ cells (open arrowheads) could be always observed (**c**-**e**), indicating dying tdT$^+$ cells being phagocytosed by EGFP$^+$ microglia. Please note, that no overlay of tdT and EGFP can be observed. Orthogonal views of selected phagocytosing EGFP$^+$ cells (white cross) were shown at the right panel. Scale bars = 20 µm.
